# How Patients with IBS Use Low FODMAP Dietary Information Provided by General Practitioners and Gastroenterologists: A Qualitative Study

**DOI:** 10.3390/nu11061313

**Published:** 2019-06-11

**Authors:** Nick Trott, Imran Aziz, Anupam Rej, David Surendran Sanders

**Affiliations:** Academic Unit of Gastroenterology, Royal Hallamshire Hospital, Sheffield Teaching Hospitals NHS Foundation Trust, Sheffield S10 2JF, UK; Imran.Aziz@sth.nhs.uk (I.A.); Anupam.Rej@sth.nhs.uk (A.R.); David.Sanders@sth.nhs.uk (D.S.S.)

**Keywords:** irritable bowel syndrome, low FODMAP diet, education delivery, nutritional information

## Abstract

There is a lack of dietitians trained to deliver the low fermentable oligosaccharides, disaccharides, monosaccharides, and polyols (FODMAP) diet (LFD) for irritable bowel syndrome (IBS). Many patients receive nutritional information from general practitioners (GPs) or gastroenterologists (GEs). Since the LFD is dietitian-led, the aim of this research was to qualitatively explore the effects of GP- and GE-delivered LFD information, in IBS self-management. Semi-structured interviews were conducted in a purposive sample of 8 people with IBS (6 female), who used the LFD as their primary treatment. Interpretive Phenomenological Analysis (IPA) was used to develop themes on the lived experience of the participant’s use of LFD information from GPs and GEs. This information was perceived as trustworthy but simplistic; often just ”food lists” with little personalisation to meet individual needs and difficult to apply in ”real life”. The information required substantial interpretation and the familial and social effects of implementation were not addressed in the materials provided. Supplementary digital resources were regarded as more practical but the participants expressed concern in relation to the validity of these materials. The findings in this study support current clinical guidelines proposed by both the National Institute for Health and Care Excellence and the British Dietetic Association that the LFD should be considered a dietitian-led only intervention.

## 1. Introduction

Irritable bowel syndrome (IBS) is a functional gastrointestinal disorder that manifests with symptoms of abdominal pain and altered bowel habit in the absence of abnormal findings on routine clinical tests explaining the symptoms [1].

The world-wide prevalence of IBS is 11.2% [2]. Patients with IBS have a significantly lower quality of life, and the economic and societal costs associated with IBS are also considerable [3,4,5]. Currently, the medical treatment of IBS is considered suboptimal and its pathophysiology is poorly understood [6]. It is thought that IBS results from abnormalities of the “gut–brain axis” (a bidirectional circuit of communication between the gut and the brain) that may involve both mucosal and neuro-inflammation [6,7,8,9,10,11]. Nutrition appears to play an important role in IBS, both in exacerbating (approximately 60% of patients) or providing relief of symptoms [12,13,14,15,16].

Over the last 10 years, dietary research has focused on the role of fermentable oligo-, di-, and mono-saccharides and polyols (FODMAPs) in relation to the induction of IBS symptoms. A substantial body of research has demonstrated the effectiveness of reducing dietary FODMAPs in treating IBS symptomology [17,18,19,20,21]. Both the National Institute for Health and Care Excellence (NICE) and the British Dietetic Association (BDA) have adopted the low FODMAP diet (LFD) into their guidelines for treatment of IBS. These guidelines emphasise the need for this approach to be delivered by a clinician/therapist who has both the necessary experience and expertise [22,23]. Thus, best practice necessitates patients who may benefit from a LFD being assessed and supported by a specialist registered dietitian (RD) with specific training in the approach [24].

The LFD is a complex three stage nutrition intervention; FODMAP restriction, reintroduction and personalisation. Specialist dietitian support ensures the approach is tailored to patients’ specific dietary needs, adequate follow-up is provided to assess its effectiveness, and FODMAPs are reintroduced to individual tolerance; thus minimising the potential risks [24,25,26,27]. These risks include alteration of the gut microbiome (specifically a reduction in the relative abundance of beneficial bifidobacteria), a reduction in dietary calcium intake and potential precipitation of disordered eating, such as orthorexia or anorexia nervosa [28,29,30,31,32,33,34].

Dietetic counselling is a central component of the research demonstrating the efficacy of the LFD [35,36,37]. This includes supporting patients to self-manage the practical application of the approach with up-to-date information and appropriate resources, including written dietary information, digital applications and online material [24,25,27]. Evidence-based guidelines for the production of effective patient information exist (see Table 1) [38,39,40]. These emphasise the importance of utilising current research and ensuring service users are integral to the development of the materials.

However, due to the increasing awareness of the LFD and a lack of suitably trained dietitians there is growing anecdotal evidence that both general practitioners (GPs) and gastroenterologists (GEs) provide patients with basic low FODMAP information [24]. Such information is often presented in the form of simple one-page lists of foods to include and avoid. Delivery of LFD information in this format has been described as deviation from best practice [41]. The aim of this research was to qualitatively explore the lived experience of how people with IBS use and apply LFD nutritional information provided by GPs and GEs to self-manage their symptoms.

## 2. Materials and Methods

### 2.1. Choice of Methodology

A qualitative research methodology, specifically Interpretive Phenomenological Analysis (IPA) was employed. IPA utilises phenomenological, hermeneutic and idiographic techniques to understand “how people ascribe meaning to their experiences in their interactions with their environment” [42]. As a functional illness the experience of IBS is highly individual and subjective in its manifestation [43]. People with IBS have vastly different experiences in their diagnostic and treatment journeys, with the somatic components of IBS inherently requiring individual interpretation [44]. Therefore, IPA is particularly suited to explore this study’s research question aiming to understand how people with IBS make sense of the dietary information they use and what this means in relation to the self-management of their symptoms [45]. A distinctive feature of IPA is its commitment to depth of analysis, and it aims to provide a detailed account of individual cases and therefore inherently requires small sample sizes [46].

### 2.2. Recruitment

A purposive, non-probability, sampling approach was used, that aimed to recruit individuals who could provide detailed information on the “lived experience” of using dietary information on the low FODMAP diet provided by GPs and GEs [47]. The participants were similar to other IBS cohorts in that they were predominately female and under 50 years of age. All participants in this study were medically diagnosed with IBS (5 via their GP and 3 via a gastroenterologist) and the principle investigator (PI) confirmed their diagnosis in reference to ROME III guidelines prior to their participation. Participants were provided with an information sheet before being interviewed and written consent was obtained.

### 2.3. Data Collection

To capture the participants’ experiences of using LFD information from GPs and GEs, semi-structured interviews were conducted by the PI. An interview schedule was produced based on perspectives highlighted from a review of the current literature, on information delivery in IBS and self-management strategies [48]. The phrasing and order of questions was iterative and developed over the interviewing process [49]. With the participant’s permission all the interviews were audio-recorded, and direct verbatim transcripts were produced. Interviews were conducted in a quiet room booked specifically for the purpose of a private, relaxed and uncluttered space likely to encourage rapport and dialogue [50].

Through discussions with clinical colleagues and the maintenance of a reflective journal, the PI attempted to bracket their own cognitions and affects in relation to the participants, during the data collection phase.

### 2.4. Data Analysis

IPA requires an iterative four-step process that includes an initial close reading and in-depth analysis of each interview transcript; noting reoccurring phrases, associations and any contradictions within the text [51]. To ensure maximal engagement with the data both the audio recordings and written transcripts of the interviews were revisited a number of times [42].

From this, emergent themes were developed, noting any possible interconnections. This process was facilitated by the use of different framing techniques including abstraction, contextualisation, polarisation and function. These formed a basis for the superordinate categories and related themes; the transcripts were frequently referred to during each stage of this activity; to further ensure the interpretative process reflected what the participant had actually said [52].

**Figure 1:**
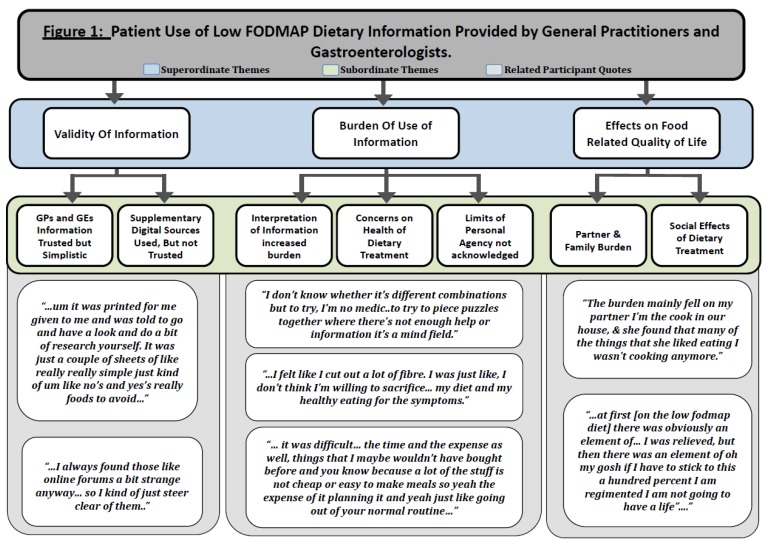
Patient use of low fermentable oligosaccharides, disaccharides, monosaccharides and polyols (low FODMAP) dietary information provided by general practitioners and gastroenterologists.

Finally, these results were ordered onto a diagram (see Figure 1) that summarises the superordinate themes, representing shared higher order qualities, and associated subordinate themes, along with relevant quotations that situate them in the participants’ actual experience [42,46].

Ethical approval was granted for this research study by the Human Ethics Committee at the University of Sheffield (approval number 012414).

## 3. Results

The purposive sample strategy initially recruited 14 participants. Upon interview it emerged that for 4 participants the LFD was not the primary treatment for their IBS, so along with two initial pilot interviews they were excluded from the analysis. All participants were either Sheffield University staff (lecturers and support staff) or under- and post-graduate students. Participants’ characteristics, including age, gender and diagnosis, are summarised (see Table 2), with the acronyms P1 to P8 being used to maintain confidentiality.

Diarrhoea predominant, was the most common IBS diagnosis amongst the cohort. The mean length of diagnosis was approximately 11 years (range 1–40 years). Six of the participants were female. All participants had received dietary information from either GPs or GEs supplemented with material from online searching.

### 3.1. Validity of Information

This was an overarching theme, recurrently emphasised by the participants. It related to an evident need to have accurate dietary information that made sense to the participants in relation to their symptoms. Information from GPs and GEs was viewed with a high degree of trust. However, the nutritional information received was equally often seen as inadequate or incomplete.

#### 3.1.1. Information from General Practitioners and Gastroenterologists Trusted but Simplistic

The participants reported a feeling of superficiality in relation to the dietary information provided by GP/GEs, the information was seen as very uniform and there was little indication of any personalisation to meet individual needs of the participants:

**“… it’s IBS you can ‘Google’ it but it probably won’t change anything, that was sort of what he said.”**

**[P3]**

There seems to have been some recognition of this by the GP/GEs, but participants were merely encouraged to assess additional material themselves. This suggests GP/GEs saw dietary information as prescriptive; advice was often reductive with blanket “allowed” and “avoid” ingredient lists:

**“…um it was printed for me given to me and was told to go and have a look and do a bit of research yourself. It was just a couple of sheets of like really simple, just kind of um like no’s and yes’s … really foods to avoid…”**

**[P4]**

#### 3.1.2. Supplementary Digital Sources Used, but Not Trusted

All the participants had supplemented the dietary information they had received from GP/GEs with digital and online resources. They often spent a significant amount of time trying to source nutritional information that had more relevance for their day to day experience:

**“…I think yeah, having confidence in the information was the important thing, having had that information [FODMAP App] that changed the way I eat, it has made a terrific difference.”**

**[P2]**

However as potential sources of information the participants expressed concern in relation to the validity of the material obtained in peer-to-peer forums. This sometimes generated a sense of distrust and could inhibit or prevent involvement with such groups:

**“…Erm, I never went into more like the chat side, just more like the NHS side, you feel like you can trust it a bit more”**

**[P3]**

### 3.2. Burden of Use of Information

This theme shadowed the participants’ sense that the dietary information they used, was in some ways incomplete. Many participants felt that information obtained from both GP/GEs and online required a process of adaption, expansion and translation to make them more personal and realistic. The resulting restrictions were often viewed as contradicting what was understood as “healthy eating”, which was again seen as an issue not addressed in the dietary information provided by GP/GEs. These issues resulted in some participants seeing the suggested alterations to their dietary habits as not just impractical but beyond the limits of their personal agency.

#### 3.2.1. Interpretation of Information Increased Burden

A common sentiment expressed by the participants centred around the dietary advice they received being often highly binary in nature, amounting to little more than “Yes/No” lists of foods. These lists were often perceived by the participants as just ingredients and did not relate to the way that they bought or consumed foods. There was no consideration of processed branded goods, food preparation, and the economic impact of the suggested dietary approaches. This was highlighted by a participant contrasting her experience with a weight loss programme:

**“…suggestions like this is a breakfast, this is a lunch … just some ideas of, like… you know that what happens with [weight loss programme] for example you don’t just get the list of foods… you get recipes and lots of things… like they do things on a budget and you just click on line and find out these things, but it was difficult to find these FODMAP foods.”**

**[P1]**

This created an additional burden to implementing the LFD. A further complication of needing to supplement the initial material was that participants reported a sense of information overload. This was particularly true when participants sort clarification and additional advice online:

**“…when you went online, … seems to be, it’s a bit like… you know, a sea of information …”**

**[P8]**

#### 3.2.2. Concerns on Health of Dietary Treatment

Participants often expressed feeling there was a contradiction between the information they received from GP/GEs and what they understood as a healthy diet. This again related to what the participants felt was a lack of personalisation in the information on the LFD:

**“…you know, a big thing like was the lack of fibre… I was just like, I don’t think I’m willing to sacrifice… my diet and my healthy eating for the symptoms.”**

**[P5]**

However, interestingly for some participants the imposed limitations caused them to be more intentional in relation to their food purchases and meal preparation. Although this was not necessarily understood as having a positive consequence, in terms of its impact on the participants social life:

**“…when you’re out it’s very very hard and when you’re buying products, erm, so I think the solution is to eat as little processed food as possible.”**

**[P6]**

#### 3.2.3. Limits of Personal Agency Not Addressed or Acknowledged

Concerns of the effect of dietary changes on health, the need to adapt information and continually monitor their “nutritional landscape” resulted in some participants questioning their ability to maintain potentially beneficial nutritional modifications over the longer term:

**“But did I find it a problem doing it..erm, I think my one, my one recollection was just thinking ‘oh my God it’s so bloody exhaustive…”**

**[P4]**

The participants awareness of the limits of their personal agency (around the use and application of dietary information from GP/GEs) appeared to contribute to their sense of restricted volition. Maintenance of a LFD was seen as unrealistic for some participants. They felt the information they received lacked both nuance in addressing their concerns and acknowledging how difficult the approach could be:

**“…what can I do… it was difficult … yeah so the time and the expense as well, things that I maybe wouldn’t have bought before and you know because a lot of the stuff is not cheap or easy to make meals so yeah the expense of it planning it and yeah just like going out of your normal routine…”**

**[P1]**

### 3.3. Effects on Food Related Quality of Life

This theme related to the dissidence participants experienced between using LFD information from GP/GEs for IBS symptom management and its effects on the social and cultural roles of food. The maintenance of social activities that centred on eating and drinking were more problematic and this had a negative impact on family dynamics.

#### 3.3.1. Partner and Family Burden

Participants related that family roles involving food were altered or lost. Nutritional behaviours that symbolised care and mutual affection were absent or changed and this impacted both the participants and their partners:

**“The burden mainly fell on my partner who suddenly found, that many of the things that she liked eating I wasn’t cooking anymore.”**

**[P6]**

**“…the impact with my partner has been that we can’t share cakes… that’s one thing we used to do, we’d go out and have coffee and half a cake each …”**

**[P2]**

#### 3.3.2. Social Effects of Dietary Treatment

Beyond immediate family members there were wider social effects of implementing LFD advice. Again, what emerged was that the information participants had received appeared to not take into account the implications and outcomes on the participants’ wider sense of community:

**“… and you feel an incredible pain because people invite you to dinner and… my friends are really great about this, but FODMAP is complicated.”**

**[P6]**

**“…there was an element of, oh my gosh, if I have to stick to this a hundred percent I am regimented. I am not going to have a life”**

**[P1]**

The information the participants received did not address or acknowledge these social effects such as the sense of being “othered” and needing to repeatedly defend their food choices:

**“… so I’m always being ‘sorry’ I’m the difficult one that needs something special…”**

**[P1]**

## 4. Discussion

A recent meta-analysis concluded that “…there is very low quality evidence that a low FODMAP diet is effective in reducing symptoms in adult IBS patients”. Issues raised included inappropriate comparator placebo arms, lack of blinding, short trial duration, and small patient cohorts [53]. However, the practice of providing basic printed literature on the LFD by GP/GEs is anecdotally recognised as widespread across the UK [24]. This pattern may have occurred as a result of insufficient dietetic support within the UK National Health Service but is not the current recommendation of existing guidelines, which recognise that the LFD is a complex elimination diet comprising three specific integrated stages [22,23]. We wonder if this practice results in reduced levels of effectiveness for this dietary intervention and reduced long-term adoption or adherence rates. This is the first study to use qualitative semi-structured interviews to explore and develop key themes on the lived experience of IBS patients being provided low FODMAP diet information from GPs and GEs.

The validity of information was important for the participants, with materials from GP/GEs being valued as a “trusted source”. However, this information was viewed as being overly simplistic, often just “food lists” with little or no personalisation to meet participants individual needs and this reduced its utility. Guidelines for the production of effective health information have been established and published [38,39,40]. This requires health information to be based on current literature and communicated in a format that patients can understand and apply; this may entail producing the information in a variety of formats. Service users should be involved in both the production and evolution of such materials through activities such as focus groups that encourage user testing [54]. It appeared the majority of the LFD information provided to the participants in this study had not been produced in line with the above recommendations.

As a consequence, the participants supplemented the information provided by GP/GEs with digital material. This was viewed as more practical, in terms of application in daily life, but equally was regarded as burdensome. It required a substantial commitment of time and often concerns were raised amongst the participants as to the validity of the information they accessed online.

The participants also voiced concerns in relation to the potential negative health effects of implementing the LFD information provided to them. Although a dietitian-led LFD broadly maintains nutrient and fibre intake, participants felt applying the materials presented to them would likely reduce the nutritional quality of their diets. Equally the elimination phase of the LFD may have detrimental effects on the human microbiome and none of the participants in this study reported being provided specific advice for the re-introduction of higher FODMAP foods to tolerance [25,55,56].

All the participants in this study re-counted negative effects of applying the LFD information on their family and social life. The act of cooking and sharing meals has been acknowledged as an important aspect of food related quality of life and family roles involving food were altered or diminished [57]. Participants described applying LFD information resulted in considerable partner burden, which is recognised in the IBS literature. Dietary interventions for IBS should aim to include partners and/or next of kin with measures to help them understand the treatment modalities [58].

Beyond immediate family members the social effects of using LFD information appeared to accentuate the participants sense of being excluded from communal events involving food. There was a sense that such events needed to be navigated rather then enjoyed. This again highlights the importance of LFD information being positioned contextually within the wider framework of an IBS sufferer’s life. Specifically, nutritional information resources that do not offer strategies of how to navigate familial and societal roles while altering dietary intake are unlikely to succeed [25].

There are several limitations to this study. As the aims of the study were to obtain in-depth data on the experience of the specific participants recruited, the findings cannot be generalised to the wider IBS population. Equally all the participants recruited were caucasian British university staff or students. Therefore inherently the experiences described, analysed and presented in the results represent a particular socio-economic demographic. Futures studies should aim to recruit from a wider range of ethnic and social backgrounds, as this would help contribute to a greater understanding of patient needs for dietary information in implementing the LFD.

In conclusion, this is the first study to explore the lived experience of IBS patients being provided low FODMAP diet information from GPs and GEs. The patients reported that the information provided was difficult to apply in “real life”. Further work is required to quantify the size of this problem. If this approach is occurring organically within the UK due to economic restraints then alternative delivery methods should be explored—such as dietitian-led low FODMAP group education, digital applications and online webinars could be developed further to support this group of patients [59,60,61]. Patients must be involved in both the production and evolution of such materials and approaches, through activities such as focus groups that encourage user testing. The findings in this study support current clinical guidelines proposed by the both by NICE and the BDA that the LFD should still be considered a second-line, dietitian-led only intervention.

## Figures and Tables

**Table 1:** Components in the Production of Effective Patient Information.

Information based on current/up-to-date literature. Communicated in a format that patients can understand and apply. Information provided in a variety of formats. Service users involved in both the production and evolution of materials.

**Table 2:** Characteristics of participants.

Participants	Age	Gender	Ethnic Origin	IBS Type	Length of Diagnosis (Years)
**P1**	24	Female	White British	IBS-D	4
**P2**	54	Female	White British	IBS-D	20
**P3**	28	Female	White British	IBS-D	1
**P4**	48	Male	White British	IBS-D	2
**P5**	28	Female	White British	IBS-D	4
**P6**	64	Male	White British	IBS-D	40
**P7**	33	Female	White British	IBS-C	6
**P8**	43	Female	White British	IBS-C	15
